# Announcement: Gordon Research Conference on Inorganic Chemistry

**DOI:** 10.1155/MBD.1996.51

**Published:** 1996


					GORDON RESEARCH CONFERENCE
ON INORGANIC CHEMISTRY
New England College, Henniker, New Hampshire,
21-26 July, 1996
David L. Thorn (DuPont), Chair; Andrew Barron (Rice University), Vice-Chair
Sunday evening, 21 July 1996,
7:30 Welcome; introductory remarks
Coordination Chemistry, part 1: A Celebration of Varied Coordination Environments
Nadine de Vries (DuPont), Discussion Leader
Frank Feher (University of California, Irvine): "Coordination Chemistry of Silsesquioxanes: The
Good, the Bad, and the Ugly"
Helmut Schwarz (Berlin): "Bond Activation by 'Bare' Transition Metal Ions: An Intersection of
Theory and Experiment"
Kit Cummins (Massachusetts Institute of Technology): "New Bond Cleavage and Atom Transfer
Reactions of Low-Coordinate Complexes"
Monday morning, 22 July
9:00 Main-group and Cluster Chemistry
Peter Dorhout (Colorado State), Discussion Leader
Steve Strauss (Colorado State University): "Fluorinated Polyhedral Heteroboranes as Weakly
Coordinating Anions"
Richard Holm (Harvard University): "Metal Clusters in Biology"
Mercouri Kanatzidis (Michigan State University): "Recent Developments in the Solid State
Chemistry of Metal Chalcogenides"
Moungi Bawendi (Massassachusetts Institute of Technology): "Semiconductor' nanocrystallites:
from isolated quantum dots to complex structures"
Monday evening, 22 July 1996
7:30 Catalysis and Catalysts
Jerry Ebner (Monsanto), Discussion Leader
Richard Kemp (Shell): "Recent Work in Polyolefin Catalysis"
Elisabeth Bordes (Compigne): "Heterogeneous Catalysis in Selective Oxidation of
Hydrocarbons: from Bulk, Crystalline to Supported, Monolayered Catalysts"
Jo Ann Canich (Exxon): "Single-Site Catalysts for Olefin Polymerization"
Tuesday morning, 23 July 1996
9:00 Surfaces, Particles, and More Clusters
Norm Herron (DuPont): "Molecular Precursors to Functional Materials"
Mike Sailor (University of California, San Diego): "Surface Chemistry of Luminescent Porous
Silicon"
Ken Klabunde (Kansas State University): "Unique Surface Chemistry of Nanoparticles of Metal
Oxides and Layered Metal Oxides"
Terry Turney (CSIRO, Australia): "Mechanical Activation of Chemical Processes"
Tuesday evening
7:30 Deposition Chemistry
Andrew Barron (Rice University), Discussion Leader
Wayne Gladfelter (University of Minnesota): "Chemical Vapor Deposition of Metallic
Compounds"
Chuck Winter (Wayne State University): "Chemistry of Precursors to Metal Nitride Films"
Tony Jones (Epichem LTD, U,K.): "Developments in Metalorganic Precursors for Vapour Phase
Epitaxy"
Wednesday morning, 24 July 1996
9:00 Coordination Chemistry, part 2: Electrons, Photons, and Radicals
Kim Dunbar (Michigan State University), Discussion Leader
Dan Nocera (Michigan State University): "Optical Supramolecules"
Dave Tyler (University of Oregon): "Cage Effects in Inorganic Radical Chemistry"
Arne Vogler (Regensberg): "Charge Transfer Excited States of Transition Metal Complexes and
Implications for Bioinorganic Chemistry and Catalysis"
Chuck Grissom (University of Utah): "The Role of Magnetic Spin in B-12 Dependent Enzymatic
and Photochemical Reactions"
Wednesday evening
7:30 Inorganic Chemistry in Biological Systems
Jacqueline Barton (California Institute of Technology): "DNA-mediated Electron Transfer with
Metallo Intercalators"
Julie Kovacs (University of Washington): "Structure and Reactivity of Models for Sulfur-Ligated
Sites of Metalloenzymes"
Scott Cunningham (DuPont): "Phytoremediation of Soils Contaminated with Heavy Metals"
Thursday morning, 25 July 1996
9:00 New Solids
Robert Haushalter (NEC Research): "Hydrothermal Synthesis of Organically Templated
Vanadium Oxide andVanadium Phosphate Solids"
Neil Bartlett (University of California, Berkeley): "The Preparation of Thermodynamically-Unstable
Transition Metal Fluorides and their Remarkable Oxidizing Properties"
Mike Whangbo (North Carolina State University): "Surface Analysis with Scanning Tunneling and
Atomic Force Microscopy"
David Mitzi (IBM): "Organic-Inorganic Perovskites"
Thursday evening
7:30 Business meeting
Francis Via (Akzo-Nobel): "The Global Research Enterprise: Leveraging Resources"
Jay Labinger (Beckmann Institute California Institute of Technology): "Science and its Critics"
52

				

